# Comparative bioequivalence of 1 mg and 4 mg tacrolimus extended-release tablets: randomized, open label, fully replicate, crossover trials in healthy adult subjects

**DOI:** 10.3389/fneph.2026.1838510

**Published:** 2026-06-08

**Authors:** Jay Kumar Sharma, Deepak Bunger, Hemant Zaveri, Alok Chaturvedi

**Affiliations:** Department of Medical Affairs, Intas Pharmaceuticals Limited, Ahmedabad, Gujarat, India

**Keywords:** bioequivalence, crossover study, extended-release, open-label, pharmacokinetic, randomized trial, tacrolimus

## Abstract

**Introduction:**

These studies were planned to establish bioequivalence between two doses (1-mg and 4-mg) of test product (tacrolimus extended-release tablets manufactured by Intas Pharmaceuticals Ltd., India) with reference product (Envarsus XR^®^ manufactured for Veloxis Pharmaceuticals, Inc., USA).

**Methods:**

These were open-label, randomized, two-treatments, four-periods, two-sequence, oral single-dose, fully-replicate, crossover trials in healthy subjects under fasting and fed conditions. The study included subjects aged 18–45 years, with a body mass index of 18.5-29.9 kg/m^2^. Bioequivalence was assessed based on key pharmacokinetic parameters (AUC, C_max_, T_max_ and t_1/2_). Safety and tolerability were assessed by monitoring adverse events (AEs), serious AEs and death.

**Results:**

The analysis set included a total of 85 (1-mg fasting), 83 (1-mg fed), 85 (4-mg fasting), and 85 (4-mg fed) subjects. Similar demographics were observed across all subjects. Results showed comparable C_max_, T_max_, AUC_0-t_, AUC_0-∞_ and t_½_ for 1-mg and 4mg mg test vs. reference product under fasting and fed conditions. The upper limit of 90% confidence intervals for within subject standard deviation for test product/within subject standard deviation for reference product (σWT/σWR), 95% upper confidence bound, and 90% confidence intervals for C_max_, AUC_0–t_, and AUC_0–∞_ were within the corresponding acceptance criteria as per EMA and met all the bioequivalence criteria.

**Conclusion:**

The developed tacrolimus test product (1-mg and 4-mg) was bioequivalent to the reference product, was well-tolerated, and provided a well-acceptable alternative to the reference product. These studies were performed in healthy individuals, further studies in transplant patient populations to confirm clinical applicability are warranted.

**CTRI number:**

These studies have not been registered on the CTRI portal. The studies are routine BA/BE studies conducted in healthy volunteers. As per prevailing regulatory guidance, CTRI registration is not mandatory for such normal BA/BE studies. Additionally, the product under evaluation is already approved worldwide, and the studies do not constitute interventional clinical trials in patients.

## 1 Introduction

In patients with end-stage organ failure, solid organ transplantation remains the standard treatment choice (1). In 2024, more than 170,000 organ transplantations were recorded globally, of which more than 63% were kidney grafts. In the same year, in India, more than 70% grafts recorded were kidney grafts of total 18,911 organ transplantations (2). However, due to shortage of donor organs, a substantial number of organ failure patients die globally. Successful grafting and increasing long-term survival of patients is the only way to offset donor organ shortage (1). Graft rejection is considered the primary cause of chronic loss of graft function. Therefore, immunosuppression in patients with organ transplantation remains an effective modality to prevent and control graft rejection (1).

Tacrolimus, a macrolide calcineurin inhibitor, is widely used as a standard of care immunosuppressive agent for preventing and treating graft rejection following solid organ transplantation, especially in kidney transplantation (3, 4). It was first approved in the USA for preventing organ rejection in patients receiving allogeneic liver in 1994, subsequently receiving kidney and heart in 1997 and 2006, respectively (5). By inhibiting T−cell activation through suppression of interleukin−2 transcription, it plays a critical role in maintaining graft survival, long−term survival, and transplant outcomes. Owing to its narrow margin between therapeutic efficacy and toxicity, tacrolimus therapy requires careful individualization of dose and therapeutic drug monitoring to ensure optimal systemic exposure (6).

Being a narrow therapeutic index (NTI) drug, relatively small variations in systemic exposure of tacrolimus may result in clinically meaningful consequences, including acute rejection, if underexposed or nephrotoxicity, neurotoxicity, and metabolic complications, if overexposed (7, 8). Additionally, it exhibits substantial pharmacokinetic variability, influenced by factors such as cytochrome P450 3A (CYP3A)–mediated metabolism, P−glycoprotein transport, formulation characteristics, and patient−specific variables (6). These characteristics necessitate a rigorous evaluation of bioequivalence, particularly when alternative formulations or generic products are introduced (6).

The introduction of generic immunosuppressants resulted in substantial reductions in treatment cost between 2008-2013, which significantly increased their utilization in the USA, with a rise in uptake approximately from 10–11% in 2009 to 76–80% by 2013 (9). The availability of generic tacrolimus reduced the overall financial burden on transplant recipients, leading to lower rates of cost-related medication nonadherence—an essential factor for long-term graft survival (9). Studies have also demonstrated that transplant patients can be safely transitioned to generic tacrolimus provided that trough drug concentrations are closely monitored (10, 11). Currently, at least ten generic tacrolimus formulations have been approved by both the European Medicines Agency (EMA) and the U.S. Food and Drug Administration (USFDA). Despite this, the market share of generic immunosuppressive drugs remains low in certain regions due to physician reluctance to prescribe generic tacrolimus. Moreover, tacrolimus is classified as an NTI drug and is approved based on standard bioequivalence criteria of 80–125% (12, 13). In contrast, the EMA recommends stricter bioequivalence requirements, specifying a narrower acceptance range of 90.00–111.11% for the area under the concentration–time curve (AUC), rather than the conventional criteria (14).

Extended−release (ER) formulations of tacrolimus have been developed to allow once−daily dosing, with the aim of improving patient adherence and maintaining more consistent drug exposure over the dosing interval (15). Improved adherence is of particular importance in transplant recipients, as non-adherence has been associated with an increased risk of graft dysfunction and loss (16). Once-daily ER formulations of tacrolimus offer several benefits over its conventional immediate release formulation and other formulations. Firstly, it delivers tacrolimus smoothly and maintains therapeutic levels over a period of time until the next dosing. Additionally, it does not cause concentration peaks associated with immediate-release formulations (17). Collectively, these offer the convenience of once-daily ER formulation over other formulations of tacrolimus available in the market. The availability of generic tacrolimus formulations offers significant potential benefits in terms of healthcare cost reduction and increased access to essential immunosuppressive therapy (18).

However, ER formulations introduce additional complexity, as differences in formulation design or drug release characteristics may affect the rate and extent of absorption, potentially affecting peak and trough concentrations (19). Consequently, demonstration of bioequivalence for tacrolimus ER products requires careful consideration of both study design and pharmacokinetic assessment. In this context, study designs capable of accurately characterizing within−subject variability and formulation performance are particularly valuable. Fully replicate crossover designs enable direct estimation of intra−subject variability for both test and reference products and are considered well suited for bioequivalence evaluation of highly variable and NTI drugs (20, 21).

We planned these BE studies among healthy individuals to reduce the variability unrelated to differences between compared test and reference products. Moreover, tacrolimus exhibits the highest rate and extent of absorption under fasting conditions. Additionally, food can significantly alter drug pharmacokinetics—such as increasing or decreasing peak concentration or total exposure—which is critical for drugs with low therapeutic indices or ER formulations. Also to assess the effects of food on the rate and extent of absorption of a drug when the drug product is administered shortly after a meal (under fed conditions), compared to administration under fasting conditions, we also conducted these comparative BE studies in healthy subjects under fed condition (22).

Therefore, the present bioequivalence studies was designed in healthy adult volunteers under fasting as well as under fed condition to compare and evaluate the bioequivalence of two doses (1-mg and 4-mg) tacrolimus ER tablets (test product, innovator tacrolimus ER tablets manufactured by Intas Pharmaceuticals Ltd., India) compared with the two doses (1-mg and 4-mg) tacrolimus ER tablets (reference product, Envarsus XR^®^ tacrolimus XR tablets manufactured for Veloxis Pharmaceuticals, Inc., North Carolina, USA). The primary objective was to assess bioequivalence based on key pharmacokinetic parameters, including the area under the concentration–time curve (AUC) and maximum observed concentration (C_max_), following single−dose administration under fasting and fed conditions. Secondary objectives included the assessment of intra−subject variability and the comparative safety and tolerability of the two products.

## 2 Methods

### 2.1 Study design and settings

**Figure 1:**
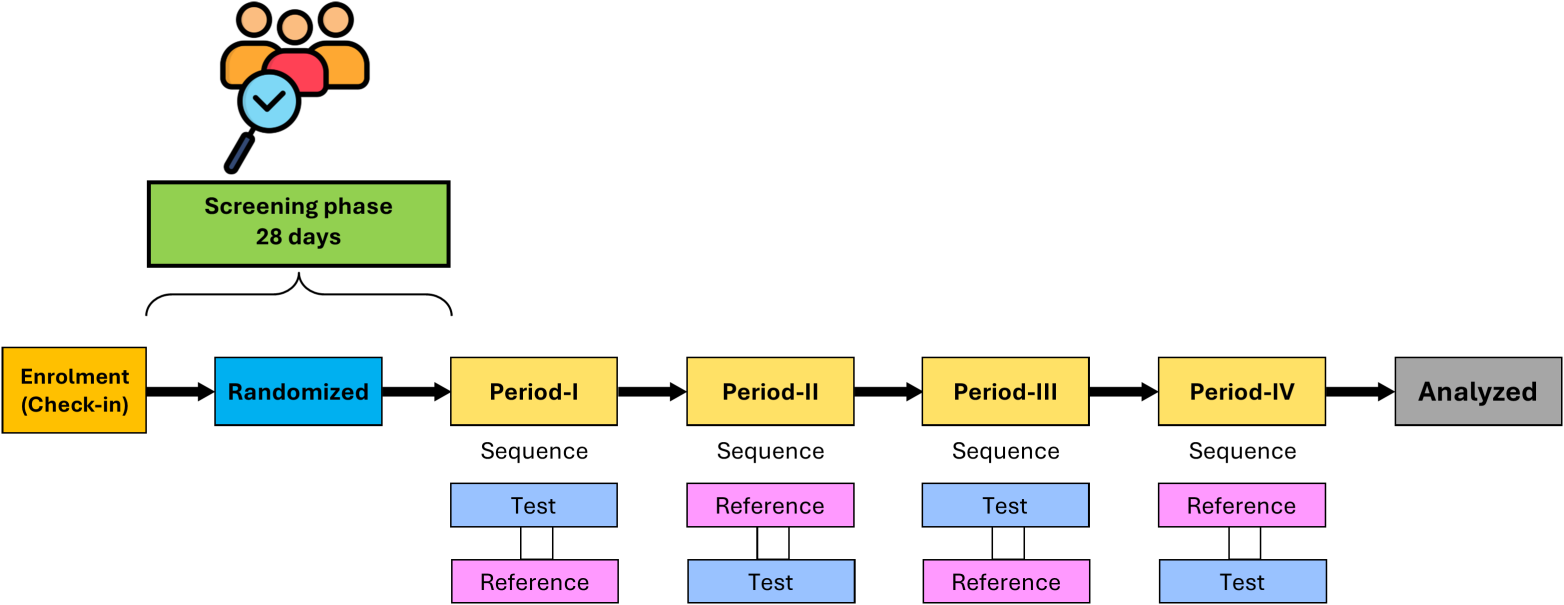
Study design.

We conducted four bioequivalence studies designed as open-label, randomized, two-treatments, four-periods, two-sequences, single oral dose, fully replicate, crossover in normal, healthy, adult, human subjects. Bioequivalence studies were conducted at the Lambda Therapeutic Research Limited, Ahmedabad, India. For each comparative bioequivalence study, based on statistical analysis and considering the possible number of dropouts/withdrawals, an estimated sample size of 90 volunteers was considered sufficient to establish bioequivalence between the test product and reference product with adequate power. Hence, for each comparative bioequivalence study, a fully replicate crossover study of 90 subjects was proposed to be included. Study design showing enrollment period (check-in), screening phase, treatment sequence, and treatment periods are presented in **Figure 1**. Briefly, volunteers were asked to report on the first day of enrollment and after completing the informed consent procedure, they underwent screening evaluations to determine eligibility within 28 days prior to the scheduled dosing of period-I. A washout period of 18–20 days was maintained between any two consecutive treatment periods.

### 2.2 Study participants

The study included normal healthy, human subjects aged 18–45 years, with a body mass index (BMI) of 18.5-29.9 kg/m^2^, who were able to understand the study procedure, and were able to provide the written informed consent. Further, they should not have any significant diseases or clinically significant abnormal findings during screening, medical history, clinical examination, laboratory evaluations, 12-lead electrocardiogram and chest X-ray (posterior-anterior view) recordings. Subjects were excluded if they had any of the exclusion criteria decided as per protocol. Detailed eligibility criteria are provided in the **Supplementary Material**.

### 2.3 Treatment and randomization

After an overnight fast of at least 10 hours, each subject received a single oral dose (1-mg or 4-mg) of either the test product (tacrolimus ER tablets manufactured by Intas Pharmaceuticals Ltd., India) or reference product (tacrolimus ER tablets manufactured for Veloxis Pharmaceuticals, Inc., North Carolina, USA) with approximately 240 ml of drinking water at ambient temperature. The test product was administered as per the randomization schedule and under open label conditions. The order of administering the test or reference products for each subject during treatment periods was determined according to the randomization schedule. In bioequivalence studies under fed conditions, subjects received a single oral dose (1-mg or 4-mg) of either the test product or the reference product at 30 minutes after serving high fat high calorie vegetarian breakfast. Other processes remained similar to that of the bioequivalence studies under fasting conditions.

### 2.4 Assessments and endpoints

Pharmacokinetic and safety measurements were carried out. Primary pharmacokinetic parameters were the maximum measured blood concentration (C_max_), area under the blood concentration vs. time curve (AUC_0-t_) in pg*h/ml, Area under the blood concentration vs. time curve (AUC_0-∞_) in pg*h/ml from time zero to infinity. Secondary pharmacokinetic parameters were the time of observing the peak concentration (T_max_) and terminal half-life (t_1/2_). These pharmacokinetic parameters were calculated for tacrolimus by non-compartmental model using Phoenix^®^ WinNonlin^®^ Version 8.1. Comparative safety and tolerability of the two products were assessed from the screening period to the end of the study through clinical examination, vital signs assessment, recording body temperature, 12-ECG, chest X-ray (posterior-anterior view), clinical laboratory examinations (e.g. hematology, biochemistry, urine analysis and immunological tests), subjective symptomatology. Adverse events (AEs), stratified by severity (mild, moderate or severe) or death were monitored and recorded.

### 2.5 Blood sampling and bioanalytical assessments

For pharmacokinetic evaluations, a total of 27 blood samples (each of 2 ml) were collected from each subject in each treatment period at different time points (pre-dose [0 hour], 0.5, 1, 1.5, 2, 2.5, 3, 3.5, 4, 4.5, 5, 5.5, 6, 6.5, 7, 8, 9, 10, 11, 12, 16, 24, 48, 72, 96, 120 and 168 hours) following product administration in each period. Precautionarily, the blood samples were kept in ice cold water bath and stored in a freezer at a temperature (ranging between −80.44 °C and −57.25 °C) till bio-analytical assessment. The blood samples were analyzed using a validated liquid chromatography-mass spectroscopy method for quantitative estimation of tacrolimus. Calibration curves with tacrolimus concentrations ranging from 40.445 pg/ml to 11978.276 pg/ml (for 1-mg studies) and 0.256 ng/ml to 99.582 ng/ml (for 4-mg studies), were plotted to determine the concentrations of tacrolimus in all blood samples.

### 2.6 Ethical considerations

These studies were carried out as per the study protocol, reviewed and approved by the ethics committee of the Care Institute of Medical Sciences (CIMS), Ahmedabad (ECR/206/Inst/GJ/2013/RR-20, protocol number: 0094-21, 0095-21, 0096–21 and 0097–21 dated 25 Nov, 24 Nov, 27 Nov and 6 Dec 2021 respectively). Studies complied with all relevant regulatory requirements for conducting clinical trials including pertinent requirements of the New Drugs & Clinical Trial Rules, 2019 of CDSCO, Ministry of Health and Family Welfare, Government of India, Declaration of Helsinki (Brazil, October 2013) and USFDA guidelines for bioequivalence studies. All the participating subjects provided written informed consents.

### 2.7 Statistical analysis

Subjects’ demographic characteristics (age, weight, height, and BMI) and pharmacokinetic parameters were summarized using descriptive statistics (mean, SD, co-efficient of variance [CV]). Analysis of variance (ANOVA) was used to estimate the within-subject SD of test and reference products. for ln-transformed pharmacokinetic parameters, 90% confidence intervals (CIs) two one-sided tests for bioequivalence (for un-scaled average bioequivalence), 95% upper bound (for scaled average bioequivalence), power and ratio analysis were performed. Using un-scaled average bioequivalence approach, bioequivalence was established for test products compared to the reference products if the 90% CI of the ratio of geometric least square means falls within the acceptance range of 80.00-125.00% for ln-transformed pharmacokinetic parameters C_max_, AUC_0-t_ and AUC_0-∞_ for Tacrolimus. Using the scaled average bioequivalence approach, bioequivalence of the test products with that of the reference product was concluded if the 95% upper confidence bound was ≤ 0 and upper limit of the 90% confidence interval for within subjects SD for test product/within subjects SD for reference product (σWT/σWR) was ≤ 2.5. Intra-subject variability of reference product and within-subject standard deviation of test and reference products were calculated and reported for lnC_max_, AUC_0-t_ and AUC_0-∞_. All statistical analyses were conducted using PROC MIXED of SAS^®^ Version 9.4 (SAS Institute Inc., USA).

## 3 Results

### 3.1 Demographic characteristics

**Figure 2:**
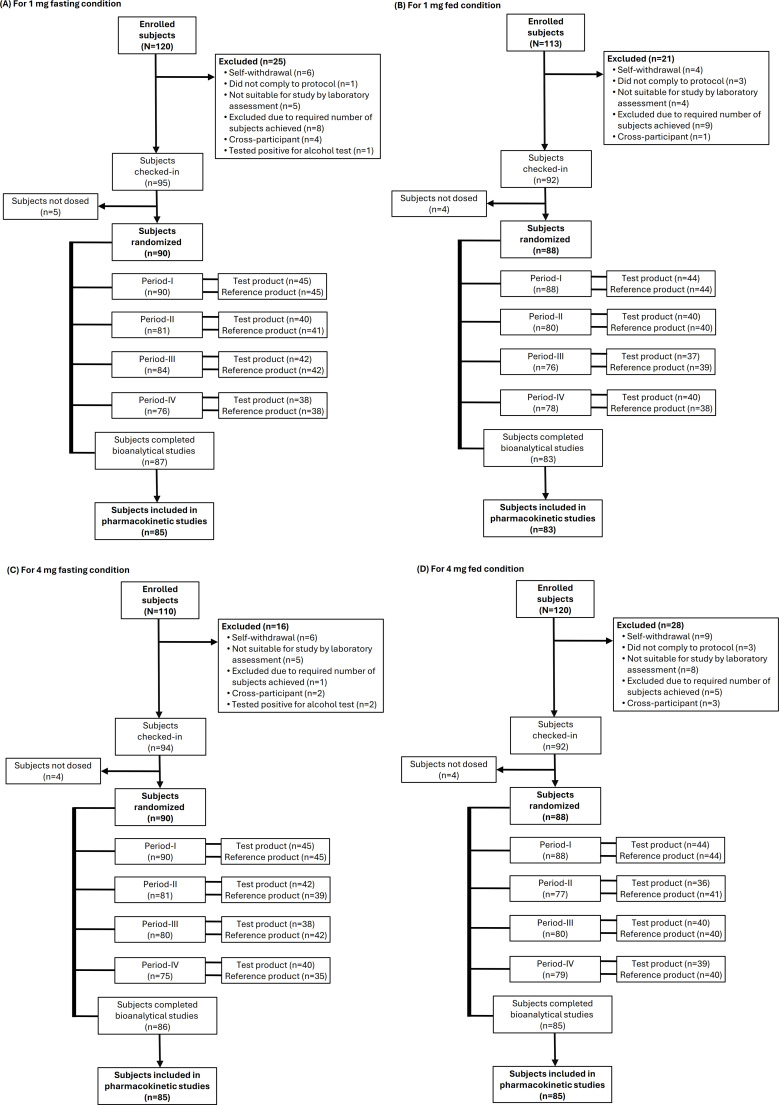
CONSORT diagram of subjects’ disposition **(A)** For 1-mg fasting condition **(B)** For 1-mg fed condition **(C)** For 4-mg fasting condition **(D)** For 4-mg fed condition.

Subjects’ disposition is presented in **Figure 2**. After considering inclusion and exclusion criteria, a total of 85, 83, 85, and 85 subjects were included in bioequivalence analysis set for 1-mg fasting condition, 1-mg fed condition, 4-mg fasting condition, and 4-mg fed condition. Subjects’ demographics characteristics are shown in **Tables 1A**, **B**. For 1-mg bioequivalence study, subjects under fasting vs. fed conditions showed similar mean age (32.5 ± 7.0 vs. 33.0 ± 6.6 years), height (165.27 ± 5.99 vs. 166.64 ± 5.92 cm), body weight (62.56 ± 8.80 vs. 62.85 ± 9.58 kg), and BMI (22.86 ± 2.65 vs. 22.60 ± 2.86 kg/m²). Comparable patterns were observed among subjects included in all dosed subjects. Similarly, in 4-mg bioequivalence study, subjects under fasting vs. fed conditions showed comparable demographic characteristics. Overall, no meaningful demographic differences were observed between fasting and fed conditions for both dose strengths.

**Table 1A:** Baseline demographics of subjects. For 1-mg tacrolimus administration.

Characteristics	Fasting condition		Fed condition	
	All dosed subjects (N = 90)	Subjects included in BE study (N = 85)	All dosed subjects (N = 88)	Subjects included in BE study (N = 83)
Age (years)	32.6 ± 6.8	32.5 ± 7.0	33.1 ± 6.5	33.0 ± 6.6
Height (cm)	165.32 ± 5.93	165.27 ± 5.99	166.91 ± 5.96	166.64 ± 5.92
Weight (km)	62.55 ± 8.67	62.56 ± 8.80	62.82 ± 9.36	62.85 ± 9.58
BMI (kg/m^2^)	22.85 ± 2.62	22.86 ± 2.65	22.52 ± 2.85	22.60 ± 2.86

**Table 1B:** For 4-mg tacrolimus administration.

Characteristics	Fasting condition		Fed condition	
	All dosed subjects (N = 90)	Subjects included in BE study (N = 85)	All dosed subjects (N = 88)	Subjects included in BE study (N = 85)
Age (years)	33.7 ± 6.2	33.7 ± 6.3	32.4 ± 6.2	32.3 ± 6.1
Height (cm)	166.13 ± 6.44	166.21 ± 6.57	165.24 ± 5.28	165.32 ± 5.23
Weight (km)	65.35 ± 9.08	65.58 ± 9.20	64.09 ± 9.46	64.23 ± 9.59
BMI (kg/m^2^)	23.68 ± 2.97	23.74 ± 3.00	23.45 ± 3.06	23.47 ± 3.10

All the values are presented as Mean ± SD.

BE, bioequivalence; BMI, body mass index; SD, standard deviation; N, number of subjects

### 3.2 Pharmacokinetic evaluation

**Figure 3:**
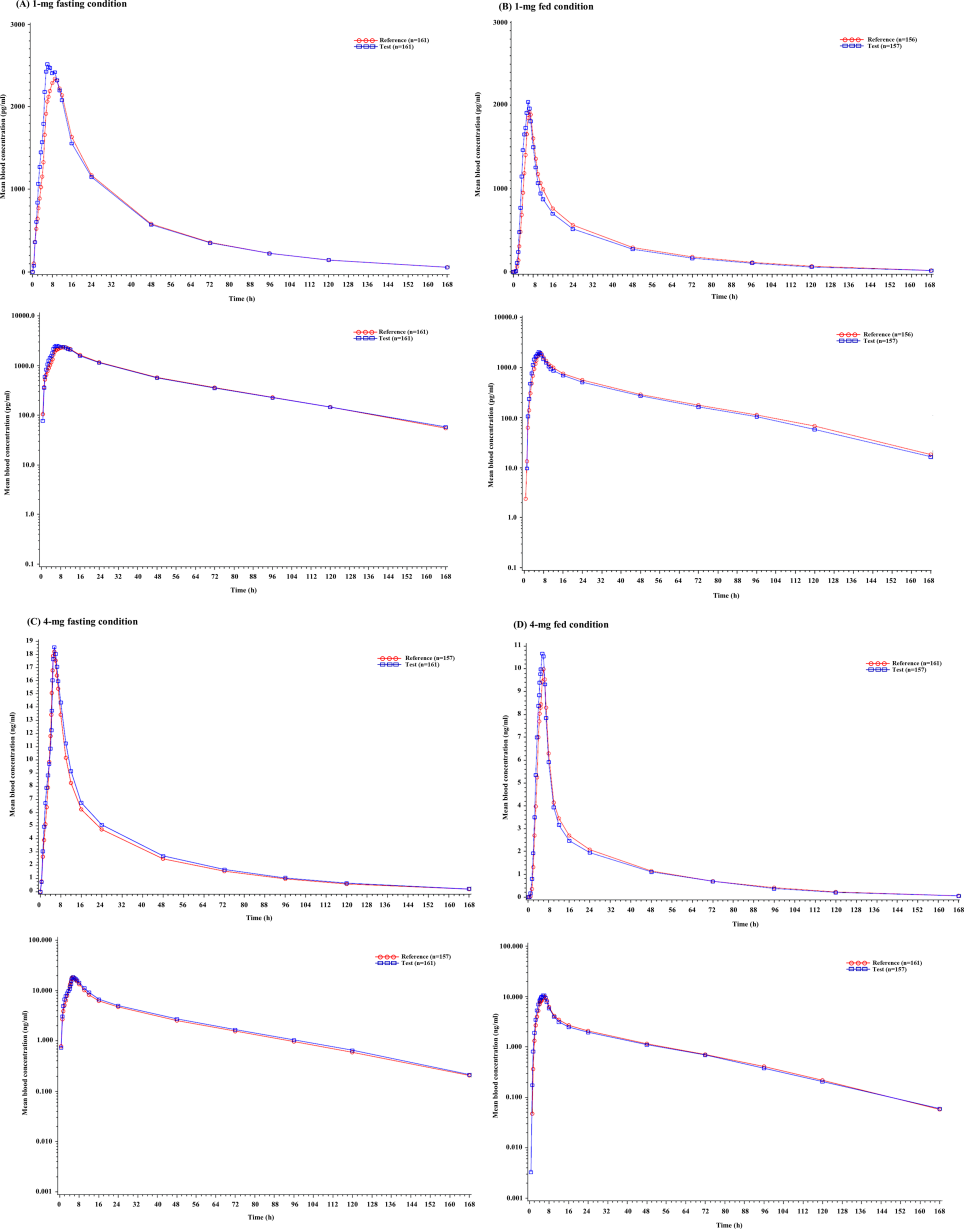
Time vs. mean blood concentration curve of tacrolimus for test and reference products **(A)** 1-mg fasting condition **(B)** 1-mg fed condition **(C)** 4-mg fasting condition **(D)** 4-mg fed condition. Curve in panel figures presents untransformed data (top) and ln-transformed data (bottom).

**Figure 3** represents the mean ± standard deviation (SD) whole blood concentration of the test and reference formulations of tacrolimus at all time points.

#### 3.2.1 1-mg study under fasting and fed conditions

Results showed comparable C_max_ (3219.37 ± 1306.23 vs. 2956.43 ± 1234.58 pg/ml), T_max_ (7.00 [2.50-16.00] vs. 9.00 [2.00-24.02] h), AUC_0-t_ (86768.08 ± 44700.09 vs. 86057.75 ± 45605.69 pg*h/ml), AUC_0-∞_ (91789.96 ± 47684.45 vs. 90900.19 ± 48454.55 pg*h/ml) and t_½_ (37.85 ± 7.04 vs. 36.83 ± 6.64 h) for 1-mg test vs. reference product, respectively, under fasting conditions (**Table 2A**). Sequence and period effects were found to be statistically insignificant (all p>0.05) for lnC_max_, lnAUC_0-t_ and lnAUC_0-∞_ (**Table 3A**). Formulation effect was found to be statistically insignificant (both p>0.05) for lnAUC_0-t_ and lnAUC_0-∞_, but statistically significant (p=0.0012) for lnC_max_. However, the upper limit of 90% CI for σWT/σWR, 95% upper confidence bound, and 90% CI met all the bioequivalence criteria (**Table 3A**).

**Table 2A:** Pharmacokinetic parameters of tacrolimus for both test and reference products. For 1-mg tacrolimus administration.

Pharmacokinetic parameters	Fasting condition (N = 85 subjects)		Fed condition (N = 83 subjects)	
	Test product (N = 161 observations)	Reference product (N = 161 observations)	Test product (N = 157 observations)	Reference product (N = 156 observations)
C_max_ (pg/mL)				
Mean ± SD (CV%)	3219.37 ± 1306.23 (40.6)	2956.43 ± 1234.58 (41.8)	2910.00 ± 1218.53 (41.9)	2656.84 ± 911.78 (34.3)
T_max_ (h)				
Median (min-max)	7.00 (2.50–16.00)	9.00 (2.00–24.02)	5.50 (1.50–24.00)	6.00 (2.50–16.00)
AUC_0-t_ (pg*h/mL)				
Mean ± SD (CV%)	86768.08 ± 44700.09 (51.5)^	86057.75 ± 45605.69 (53.0)	42287.71 ± 21723.17 (51.4)^#^	44041.04 ± 24531.59 (55.7)^#^
AUC_0-∞_ (pg*h/mL)				
Mean ± SD (CV%)	91789.96 ± 47684.45 (51.9)^	90900.19 ± 48454.55 (53.3)	45551.84 ± 22698.43 (49.8)^#^	47452.34 ± 25902.09 (54.6)^#^
t_½_ (h)				
Mean ± SD (CV%)	37.85 ± 7.04 (18.6)^	36.83 ± 6.64 (18.0)	36.62 ± 7.54 (20.6)^#^	37.01 ± 6.65 (18.0)^#^

**Table 2B:** For 4-mg tacrolimus administration.

Pharmacokinetic parameters	Fasting condition (N = 85 subjects)		Fed condition (N = 85 subjects)	
	Test product (N = 161 observations)	Reference product (N = 157 observations)	Test product (N = 157 observations)	Reference product (N = 161 observations)
C_max_ (ng/mL)				
Mean ± SD (CV%)	21.11 ± 7.65 (36.2)	20.81 ± 7.84 (37.7)	16.71 ± 6.47 (38.7)	16.44 ± 6.41 (39.0)
T_max_ (h)				
Median (min-max)	5.50 (2.00–12.00)	5.50 (2.00–12.03)	5.00 (2.00–12.00)	5.00 (2.00–12.00)
AUC_0-t_ (ng*h/mL)				
Mean ± SD (CV%)	430.80 ± 220.44 (51.2)^	400.74 ± 198.10 (49.4)^@^	173.10 ± 110.63 (63.9)^$^	175.23 ± 100.59 (57.4)^
AUC_0-∞_ (ng*h/mL)				
Mean ± SD (CV%)	454.85 ± 230.07 (50.6)^	424.78 ± 208.86 (49.2)^@^	191.89 ± 117.88 (61.4)^$^	193.89 ± 105.57 (54.5)^
_½_ (h)				
Mean ± SD (CV%)	35.52 ± 6.60 (18.6)^	35.91 ± 7.44 (20.7)^@^	34.90 ± 8.57 (24.6)^$^	35.03 ± 8.87 (25.3)^

^ N = 160 observations, ^#^ N=155 observations, ^@^ N=156 observations, ^$^ N=154 observations.

C_max_*_,_* Maximum measured blood concentration; T_max_, Time of the maximum measured blood concentration; AU*C*_0-t_, Area under the blood concentration versus time curve from time zero to the last measurable concentration as calculated by linear trapezoidal method; AUC_0-∞_, Area under the blood concentration versus time curve from time zero to infinity; _½_, Terminal half-life; Test product , Tacrolimus extended-release tablets manufactured by Intas Pharmaceuticals limited; India, Reference product = tacrolimus XR tablets manufactured for Veloxis Pharmaceuticals, Inc., Cary, North Carolina.

CV, coefficient of variance; SD, standard deviation.

**Table 3A:** Relative bioavailability of test and reference products. For 1-mg tacrolimus under fasting condition (N = 85).

Parameters	Geometric least square means			90% CI	Upper limit of 90% CI for σWT/σWR	95% upper confidence bound	Power (%)	ANOVA (p-value)		
	Test product (161 observations)	Reference product (161 observations)	Ratio (T/R) %					Formulation	Sequence	Period
lnC_max_	2980.081	2707.461	110.1	104.90–115.50	1.164	-0.0512	100.0	0.0012	0.6911	0.6195
lnAUC_0-t_	76028.890^	74274.684	102.4	96.81–108.23	1.187	-0.0916	100.0	0.4902	0.8876	0.0909
lnAUC_0-∞_	80613.795^	78752.487	102.4	97.10–107.91	1.200	-0.0807	100.0	0.4656	0.8926	0.0737

Comparable C_max_ (2910.00 ± 1218.53 vs. 2656.84 ± 911.78 pg/ml), T_max_ (5.50 [1.50-24.00] vs. 6.00 [2.50-16.00] h), AUC_0-t_ (42287.71 ± 21723.17 vs. 44041.04 ± 24531.59 pg*h/ml), AUC_0-∞_ (45551.84 ± 22698.43 vs. 47452.34 ± 25902.09 pg*h/ml) and t_½_ (36.62 ± 7.54 vs. 37.01 ± 6.65 h) were observed for 1-mg test vs. reference product, respectively, under fed conditions (**Table 2A**). Sequence effect was found to be statistically insignificant (p>0.05) for ln-transformed pharmacokinetic parameters lnC_max_, lnAUC_0-t_ and lnAUC_0-∞_ (**Table 3B**). Formulation and period effects were found to be statistically insignificant (both p>0.05) for lnAUC_0-t_ and lnAUC_0-∞_, but statistically significant (p<0.05) for lnC_max_. However, the upper limit of 90% CI for σWT/σWR, 95% upper confidence bound, and 90% CI met all the bioequivalence criteria (**Table 3B**).

**Table 3B:** For 1-mg tacrolimus under fed condition (N = 83).

Parameters	Geometric least square means			90% CI	Upper limit of 90% CI for σWT/σWR	95% upper confidence bound	Power (%)	ANOVA (p-value)		
	Test product (157 observations)	Reference product (156 observations)	Ratio (T/R) %					Formulation	Sequence	Period
lnC_max_	2693.424	2490.847	108.1	102.72– 113.83	1.774	-0.0202	100.0	0.0132	0.6546	0.0002
lnAUC_0-t_	37858.103	38728.200	97.8	93.98– 101.68	1.133	-0.0339	100.0	0.3399	0.6649	0.1278
lnAUC_0-∞_	41077.190	42008.797	97.8	94.19– 101.51	1.181	-0.0288	100.0	0.3219	0.6598	0.1220

#### 3.2.2 4-mg study under fasting and fed conditions

Results showed very similar C_max_ (21.11 ± 7.65 vs. 20.81 ± 7.84 ng/ml), T_max_ (5.50 [2.00-12.00] vs. 5.50 [2.00-12.03] h), AUC_0-t_ (430.80 ± 220.44 vs. 400.74 ± 198.10 ng*h/ml), AUC_0-∞_ (454.85 ± 230.07 vs. 424.78 ± 208.86 ng*h/ml) and t_½_ (35.52 ± 6.60 vs. 35.91 ± 7.44 h) for 4-mg test vs. reference product, respectively, under fasting conditions (**Table 2B**). Sequence effect was found to be statistically insignificant (all p>0.05) for lnC_max_, lnAUC_0-t_ and lnAUC_0-∞_ (**Table 3C**). Formulation and period effects were found to be statistically insignificant (both p>0.05) for lnC_max_, but statistically significant (p<0.05) for lnAUC_0-t_ and lnAUC_0-∞_. However, the upper limit of 90% CI for σWT/σWR, 95% upper confidence bound, and 90% CI met all the bioequivalence criteria (**Table 3C**).

**Table 3C:** For 4-mg tacrolimus under fasting condition (N = 85).

Parameters	Geometric least square means			90% CI	Upper limit of 90% CI for σWT/σWR	95% upper confidence bound	Power (%)	ANOVA (p-value)		
	Test product (161 observations)	Reference product (157 observations)	Ratio (T/R) %					Formulation	Sequence	Period
lnC_max_	19.534	19.227	101.6	97.44–105.94	1.259	-0.0452	100.0	0.5313	0.9046	0.5641
lnAUC_0-t_	376.061^	351.336^@^	107.0	102.02–112.30	1.087	-0.0569	100.0	0.0201	0.8048	0.0070
lnAUC_0-∞_	398.926^	373.728^@^	106.7	101.95–111.76	1.073	-0.0533	100.0	0.0200	0.7918	0.0071

Almost similar C_max_ (16.71 ± 6.47 vs. 16.44 ± 6.41 ng/ml), T_max_ (5.00 [2.00-12.00] vs. 5.00 [2.00-12.00] h), AUC_0-t_ (173.10 ± 110.63 vs. 175.23 ± 100.59 ng*h/ml), AUC_0-∞_ (191.89 ± 117.88 vs. 193.89 ± 105.57 ng*h/ml) and t_½_ (34.90 ± 8.57 vs. 35.03 ± 8.87 h) were observed for 4-mg test vs. reference product, respectively, under fed conditions (**Table 2B**). Formulation and Sequence effects were found to be statistically insignificant (all p>0.05) for ln-transformed pharmacokinetic parameters lnC_max_, lnAUC_0-t_ and lnAUC_0-∞_ (**Table 3D**). Period effect was found to be statistically insignificant (p>0.05) for lnAUC_0-t_ and lnAUC_0-∞_, but statistically significant (p<0.05) for lnC_max_. However, the upper limit of 90% CI for σWT/σWR, 95% upper confidence bound, and 90% CI met all the bioequivalence criteria (**Table 3D**).

**Table 3D:** For 4-mg tacrolimus under fed condition (N = 85).

Parameters	Geometric least square means			90% CI	Upper limit of 90% CI for σWT/σWR	95% upper confidence bound	Power (%)	ANOVA (p-value)		
	Test product (157 observations)	Reference product (161 observations)	Ratio (T/R) %					Formulation	Sequence	Period
lnC_max_	15.563	15.265	102.0	96.96– 107.20	1.224	-0.0384	100.0	0.5231	0.9008	0.0054
lnAUC_0-t_	145.340^$^	148.591^	97.8	94.29– 101.46	1.248	-0.0223	100.0	0.3178	0.8197	0.0.0615
lnAUC_0-∞_	164.121^$^	167.508^	98.0	94.63– 101.45	1.198	-0.0184	100.0	0.3311	0.7848	0.0623

^ N = 160 observations, ^@^ N=156 observations, ^$^ N=154 observations.

Significance was considered if p-value<0.05

lnC_max_, ln-transformed maximum measured blood concentration, T_max_, Time of the maximum measured blood concentration, lnAU*C*_0-t_ , ln-transformed area under the blood concentration versus time curve from time zero to the last measurable concentration as calculated by linear trapezoidal method, lnAUC_0-∞ ,_ ln-transformed area under the blood concentration versus time curve from time zero to infinity, σWT = within-subject SD of test product, σWR , within-subject SD of reference product, Test product , Tacrolimus extended-release tablets manufactured by Intas Pharmaceuticals limited, India, Reference product , tacrolimus XR tablets manufactured for Veloxis Pharmaceuticals, Inc., Cary, North Carolina.

ANOVA, analysis of variance; CI, confidence interval; SD, standard deviation.

### 3.3 Safety assessment

Incidence and types of AEs are presented in **Table 4**. Following 1-mg tacrolimus administration under fasting conditions, overall, 21 AEs were reported: 11 AEs in the subjects receiving reference product; 10 AEs in the subjects receiving test product. Of these, 19 AEs were mild, and two were moderate in nature. Similarly, under fed conditions, 21 AEs were reported: 12 AEs in the subjects receiving reference product; nine AEs in the subjects receiving test product. Of these 21 AEs, one AE was serious (viral encephalitis), 19 AEs were mild, and one was moderate. Viral encephalitis (serious AE) was reported in the patient who received 1-mg reference product. There were no other serious AEs reported during the conduct of the study.

**Table 4A:** Results of incidence of AEs after administration of test and reference product. 1-mg under fasting condition.

AEs	Test product (N = 87)		Total	Reference product (N = 88)		Total
	Related	Not related		Related	Not related	
Investigations						
Blood creatinine increased	1 (1.15)	–	1 (1.15)	–	–	–
Platelet counts decreased	–	1 (1.15)	1 (1.15)	–	–	–
Alanine aminotransferase increased	–	–	–	1 (1.14)	–	1 (1.14)
Blood bilirubin increased	–	–	–	1 (1.14)	1 (1.14)	2 (2.27)
White blood cell counts increased	–	–	–	–	1 (1.14)	1 (1.14)
Aspartate aminotransferase increased	–	–	–	–	1 (1.14)	1 (1.14)
Transaminase increased	–	–	–	1 (1.14)	–	1 (1.14)
Injury, poisoning and procedural complications						
Limb injury	–	1 (1.15)	1 (1.15)	–	–	–
Injury	–	–	–	–	1 (1.14)	1 (1.14)
Thermal burn	–	–	–	–	1 (1.14)	1 (1.14)
Gastrointestinal disorders						
Abdominal pain	1 (1.15)	1 (1.15)	2 (2.30)	1 (1.14)	–	1 (1.14)
Diarrhea	–	–	–	1 (1.14)	–	1 (1.14)
Skin and subcutaneous tissue disorders						
Skin infection	–	2 (2.30)	2 (2.30)	–	1 (1.14)	1 (1.14)
Rash pruritic	–	1 (1.15)	1 (1.15)	–	–	–
General disorders and administration site conditions						
Pain	–	1 (1.15)	1 (1.15)	–	–	–
Ear and labyrinth disorders						
Otorrhea	–	1 (1.15)	1 (1.15)	–	–	–

**Table 4B:** 1-mg under fed condition.

AEs	Test product (N = 86)		Total	Reference product (N = 85)		Total
	Related	Not related		Related	Not related	
Investigations						
Alanine aminotransferase increased	–	1 (1.16)	1 (1.16)	–	–	–
White blood cell counts increased	–	1 (1.16)	1 (1.16)	–	2 (2.35)	2 (2.35)
Neutrophil count increased	–	1 (1.16)	1 (1.16)	–	1 (1.18)	1 (1.18)
Blood bilirubin increased	–	1 (1.16)	1 (1.16)	–	–	–
Injury, poisoning and procedural complications						
Injury	–	2 (2.33)	2 (2.33)	–	2 (2.35)	2 (2.35)
Gastrointestinal disorders						
Abdominal pain	–	1 (1.16)	1 (1.16)	–	–	–
Diarrhea	–	1 (1.16)	1 (1.16)	–	–	–
Vomiting	–	–	–	2 (2.35)	1 (1.18)	3 (3.53)
Vascular disorders						
Dizziness	1 (1.16)	–	1 (1.16)	–	–	–
General disorders and administration site conditions						
Pyrexia	–	–	–	–	2 (2.35)	2 (2.35)
Infections and infestations						
Viral encephalitis	–	–	–	–	1 (1.18)	1 (1.18)
Musculoskeletal and connective tissue disorders						
Ankle fracture	–	–	–	–	1 (1.18)	1 (1.18)

**Table 4C:** 4-mg under fasting condition.

AEs	Test product (N = 88)		Total	Reference product (N = 87)		Total
	Related	Not related		Related	Not related	
Investigations.						
Blood bilirubin increased	1 (1.14)	1 (1.14)	2 (2.27)	–	2 (2.30)	2 (2.30)
Platelet counts decreased	–	1 (1.14)	1 (1.14)	–	–	–
Aspartate aminotransferase increased	–	1 (1.14)	1 (1.14)	–	1 (1.15)	1 (1.15)
White blood cell counts increased	–	1 (1.14)	1 (1.14)	–	–	–
Hemoglobin decreased	–	1 (1.14)	1 (1.14)	–	–	–
Alanine aminotransferase increased	–	–	–	–	2 (2.30)	2 (2.30)
Red blood cell counts decreased	–	–	–	2 (2.30)	–	2 (2.30)
Injury, poisoning and procedural complications						
Skin abrasion	–	–	–	–	2 (2.30)	2 (2.30)
Injury	–	2 (2.27)	2 (2.27)	–	1 (1.15)	1 (1.15)
Gastrointestinal disorders						
Vomiting	1 (1.14)	–	1 (1.14)	–	–	–
Nausea	–	–	–	1 (1.15)	–	1 (1.15)
Abdominal pain	–	–	–	1 (1.15)	–	1 (1.15)
Musculoskeletal and connective tissue disorders						
Neck pain	–	1 (1.14)	1 (1.14)	–	–	–
General disorders and administration site conditions						
Ulcer	–	1 (1.14)	1 (1.14)	–	–	–
Pain	–	–	–	1 (1.15)	2 (2.30)	3 (3.45)
Respiratory, thoracic and mediastinal disorders						
Musculoskeletal chest pain	–	1 (1.14)	1 (1.14)	–	–	–
Nervous system disorders						
Headache	–	–	–	–	1 (1.15)	1 (1.15)
Infections and infestations						
Upper respiratory tract infection	–	–	–	–	1 (1.15)	1 (1.15)
Renal and urinary disorders						
Dysuria	–	–	–	1 (1.15)	–	1 (1.15)

For 4-mg tacrolimus under fasting conditions, 30 AEs were reported: 18 AEs in the subjects receiving reference product; 12 AEs in the subjects receiving test product. Of these, 28 AEs were mild, and two were moderate in nature. Similarly, under fed conditions, 18 AEs were reported: 11 AEs in the subjects receiving reference product; seven AEs in the subjects receiving test product. All AEs were mild in nature. No serious AEs or deaths were experienced during the study. Most AEs were related to investigations, gastrointestinal disorders, skin disorders, general disorders, eye disorders, and respiratory disorders. All the AEs were resolved with or without medication support.

**Table 4D:** 4-mg under fed condition.

AEs	Test product (N = 86)		Total	Reference product (N = 87)		Total
	Related	Not related		Related	Not related	
Investigations						
Blood bilirubin increased	–	1 (1.16)	1 (1.16)	–	–	–
Aspartate aminotransferase increased	–	–	–	–	1 (1.15)	1 (1.15)
White blood cell counts increased	–	–	–	–	1 (1.15)	1 (1.15)
Hepatic enzyme increased	–	–	–	–	1 (1.15)	1 (1.15)
Alanine aminotransferase increased	–	4 (4.65)	4 (4.65)			
Neutrophil count increased	–	–	–	–	1 (1.15)	1 (1.15)
Blood creatinine increased	–	–	–	–	2 (2.30)	2 (2.30)
Injury, poisoning and procedural complications						
Skin abrasion	–	–	–	–	1 (1.15)	1 (1.15)
Gastrointestinal disorders						
Vomiting	1 (1.16)	–	1 (1.16)	1 (1.15)	–	1 (1.15)
Immune system disorders						
Urticaria	–	–	–	1 (1.15)	–	1 (1.15)
General disorders and administration site conditions						
Chest pain	–	1 (1.16)	1 (1.16)	1 (1.15)	–	1 (1.15)
Eye disorders						
Conjunctival hyperemia	–	–	–	–	1 (1.15)	1 (1.15)

Values are presented as number and (%) of events.

Calculation of AEs, Total number of AEs*100/Total number of subjects who had consumed either test product or reference product at least once during the conduct of the study.

AEs, adverse events; N , number of subjects included in safety analysis.

## 4 Discussion

The present single-dose, randomized, open-label, cross-over BE studies evaluated 1-mg and 4-mg tacrolimus ER tablets under fasting and fed conditions and confirmed that the pharmacokinetic parameters of the test formulations were comparable to those of the reference product. The bioequivalence shown in our study implies that similar tacrolimus exposure is achieved when individuals are switched between these tacrolimus products.

Consistent demographic characteristics between treatment periods minimized potential confounding effects. Across both strengths, the 90% CIs for C_max_, AUC_0–t_, and AUC_0–∞_ met all regulatory bioequivalence requirements, despite isolated statistically significant period or formulation effects in lnC_max_ or lnAUC values. These results indicated that the 1-mg and 4-mg ER test products of tacrolimus met the regulatory definition of bioequivalence to the reference product in accordance with EMA guidelines. These findings align with known tacrolimus variability and support interchangeability between the products. Published evaluations of tacrolimus XR tablets have similarly demonstrated predictable pharmacokinetic behavior, with controlled absorption and reduced peak–trough fluctuations in diverse patient populations.

The observed pharmacokinetic characteristics in our studies are consistent with earlier comparative studies demonstrating that tacrolimus ER formulations maintain extended absorption and reduced fluctuation, even in rapid metabolizers or stable renal transplant recipients (16, 17). Tremblay et al. reported that tacrolimus XR tablets provide an improved bioavailability and a smoother pharmacokinetic profile compared with immediate release tacrolimus, with a 30% required dose reduction—evidence that parallels the stable and reproducible exposure outcomes seen in the present analysis (17). Similarly, Rostaing et al. confirmed long-term comparable efficacy and safety of once daily ER tacrolimus vs. twice daily tacrolimus, with lower total daily dose requirements but consistent trough concentrations over a 24-months period (16). Together, these studies reinforce that the ER mechanism reliably moderates tacrolimus exposure while achieving bioequivalence, as demonstrated in our single dose crossover studies.

The findings of our studies are in agreement with previously published literature on tacrolimus ER formulations. Studies evaluating tacrolimus XR tablets and other modified-release tacrolimus products have similarly reported extended absorption phases with median T_max_ values ranging from 4 to 8 hours and half-life approximating 34–40 hours, depending on population and conditions (17, 23). Prior comparative pharmacokinetic studies have demonstrated that tacrolimus ER formulations maintain bioequivalence across different food conditions despite known food related reductions in C_max_, a phenomenon also observed in the present 1-mg and 4-mg datasets. Furthermore, the relatively lower C_max_ variability seen with tacrolimus XR tablets in historical reports aligns with the comparable σ_WT_/σ_WR_ ratios achieved here, supporting consistent drug exposure and controlled release characteristics.

As there is very small margin between a safe and a lethal dose, drugs with a narrow margin of safety that may not be satisfied with current general standard bioequivalence range of 80–125% must be selected very critically. EMA suggest 90.00–111.11% acceptance criteria for AUC for drug products with NTI (14). Moreover, when C_max_ is of particular importance considering safety, efficacy, or drug level monitoring, all these parameters should also fall within 90.00–111.11%. In our studies, using scaled bioequivalence approach, we considered 90.00–111.11% as the acceptance range for 90% CI for the AUC and C_max_. Results of the studies indicated that 90% CIs for C_max_, AUC_0–t_, and AUC_0–∞_ were within the corresponding acceptance criteria as per EMA.

The safety findings reported in our studies—predominantly mild AEs with no serious AEs or death—corroborates the established tolerability of tacrolimus ER. Across all study arms, AEs were predominantly mild and self-limiting, with no serious AEs were reported. These findings reflect earlier clinical pharmacology reports showing that healthy volunteers typically experience only transient gastrointestinal or neurological symptoms following single-dose tacrolimus administration (24, 25). Bekersky et al. documented that tacrolimus is generally well tolerated in single-dose studies, with transient gastrointestinal and neurological symptoms being the most common AEs (25). Notably, the similarity in AE incidence between test and reference products further strengthens the evidence for clinical interchangeability. Though viral encephalitis was reported in one patient following administration of 1-mg reference product under fed condition, results showed no new safety concerns, and AE profiles were similar between the test and reference formulations, reinforcing their comparable tolerability.

This study is strengthened by its robust sample sizes, comprehensive PK sampling, and replicated assessment across fasting and fed conditions for two strengths. Additionally, validated whole-blood tacrolimus quantification supports high analytical reliability. Nonetheless, the studies have some limitations also. As a single-dose investigation in healthy subjects, it does not reflect steady-state kinetics or clinical performance in transplant patients, who may have altered physiology or higher pharmacokinetic variability. Prior population pharmacokinetic analyses have shown that transplant recipients may require individualized dosing due to metabolic, genetic, or comorbidity factors—highlighting a gap that future studies should address.

## 5 Conclusion

The tacrolimus 1-mg and 4-mg ER tablets (manufactured by Intas Pharmaceuticals Ltd., Gujarat, India) was bioequivalent to tacrolimus XR tablets (manufactured for Veloxis Pharmaceuticals, Inc., North Carolina, USA). Further, test products were found well-tolerated and demonstrated a well-acceptable safety profile. Switching treatment to generic tacrolimus product may reduce the treatment cost and economic burden. As these comparative bioequivalence studies were performed in healthy individuals, results should be interpreted cautiously when extrapolating to transplant patients. This also warrants further study in transplant patient populations to confirm clinical applicability.

## Data Availability

The original contributions presented in the study are included in the article/**Supplementary Material**. Further inquiries can be directed to the corresponding author.
