# Supplementary material for: Comparative bioequivalence of 1 mg and 4 mg tacrolimus extended-release tablets: randomized, open label, fully replicate, crossover trials in healthy adult subjects
Source: Front Nephrol. 2026 Jun 8;6:1838510. doi: 10.3389/fneph.2026.1838510 (PMC13284695; doi:10.3389/fneph.2026.1838510)
Supplement: Supplementary file 1 [file DataSheet1.docx]

Supplementary Material

*Inclusion criteria*

The subjects were selected based on the following inclusion criteria per the protocol:

- Normal, healthy, adult, human subjects between 18 and 45 years of age (both inclusive)
- Having a body mass index between 18.5 and 29.9 (both inclusive) kg/m^2^
- Not having significant diseases or clinically significant abnormal findings during screening, medical history, clinical examination, laboratory evaluations, 12-lead ECG and chest X-ray (posterior-anterior view) recordings
- Able to understand and comply with the study procedures, in the opinion of the principal investigator
- Able to give voluntary written informed consent for participation in the study
- Female subjects should be surgically sterilized at least 6 months prior to study participation or if they had childbearing potential, then they should be willing to use a suitable and effective double barrier contraceptive method or intra uterine device during the study along with confirmed negative serum pregnancy test

*Exclusion criteria*

The subjects were excluded based on the following exclusion criteria per the protocol:

- With known hypersensitivity or idiosyncratic reaction to tacrolimus or any related drug or any of its excipients
- With the history or presence of any disease or condition which might compromise the haemopoietic, renal, hepatic, endocrine, pulmonary, central nervous, cardiovascular, immunological, dermatological, gastrointestinal or any other body system
- If they ingested any medicine (prescribed medication and over the counter medication including herbal remedies, CYP3A inhibitors/inducers (including but not limited to telaprevir, boceprevir, ritonavir, ketoconazole, itraconazole, voriconazole, clarithromycin rifampin, rifabutin), aminoglycosides, ganciclovir, amphotericin B, cisplatin, nucleotide reverse transcriptase inhibitors, protease inhibitors, potassium-sparing diuretics, ACE inhibitors, angiotensin receptor blockers) at any time within 14 days prior to dosing in period-I
- Having history and presence of seizure
- The presence of clinically significant abnormal laboratory values during screening
- Difficulty in swallowing oral solid dosage form like tablet
- Any history or presence of asthma (including aspirin induced asthma) or nasal polyp or non-steroidal anti-inflammatory drugs (i.e. ibuprofen, aspirin) induced urticaria
- Consumption of grapefruits or grapefruit products within 72 hours prior to dosing of period-I
- A recent history of harmful use of alcohol (less than 2 years), i.e. alcohol consumption of more than 14 standard drinks per week for men and more than 07 standard drinks per week for women (A standard drink is defined as 360 ml of beer or 150 ml of wine or 45 ml of 40% distilled spirits, such as rum, whisky, brandy etc.) or consumption of alcohol or alcoholic products within 48 hours prior to receiving study medicine of period-I
- Smokers, who smoke 10 or more than 10 cigarettes/day or inability to abstain from smoking during the study
- Use of any recreational drugs or history of drug addiction or testing positive in pre-study drug scans
- History or presence of seizure or psychiatric disorders.
- A history of difficulty with donating blood.
- Donation of blood (1 unit or 350 ml) within a period of 90 days prior to the first dose of study medication.
- Receipt of an investigational medicinal product or participation in a drug research study within a period of 90 days prior to the first dose of study medication. (if investigational medicinal product is received within 90 days where there is no blood loss except safety laboratory testing, subject can be included considering 10 half-lives duration of investigational medicinal product received
- A positive hepatitis screen including hepatitis B surface antigen and/or HCV antibodies
- A positive test result for HIV (1 and/or 2)
- An unusual diet, for whatever reason (e.g. low-sodium), for four weeks prior to receiving the study drug in Period-I. In any such case, subject selection will be at the discretion of the principal investigator
- Nursing mothers
